# Open-Source Anatomical Illustration for Digital Back Pain Documentation: Development and Feasibility Evaluation

**DOI:** 10.2196/75737

**Published:** 2026-10-15

**Authors:** Yanick Xavier Lukic, Sonja Schläpfer, Alessandro Holler, Giovanni Colacicco, Dennis Baumli, Leana Wirth, Steven Häsler, Christoph J Laux, Reto Sutter, Georg Wilhelm Kajdi, Benedikt Herzog, Sebastiano Caprara, Claudia M Witt, Philipp Ackermann

**Affiliations:** 1 Institute of Computer Science, School of Engineering, Zurich University of Applied Sciences, Steinberggasse 13, Winterthur, 8400, Switzerland; 2 School of Medicine, University of St.Gallen, St.Gallen, Switzerland; 3 Complementary and Integrative Digital Health, Institute of Primary Care, University of Zurich and University Hospital Zurich, Zurich, Switzerland; 4 Institute for Design Research, Zurich University of the Arts, Zurich, Switzerland; 5 Quaint GmbH, Zurich, Switzerland; 6 Institute of Anatomy, University of Zurich, Zurich, Switzerland; 7 University Spine Center Zurich, Balgrist University Hospital, University of Zurich, Zurich, Switzerland; 8 Department of Radiology, Balgrist University Hospital, University of Zurich, Zurich, Switzerland; 9 Digital Medicine Unit, Balgrist University Hospital, Zurich, Switzerland

**Keywords:** back pain documentation, anatomical illustration, digital health, Systematized Nomenclature of Medicine Clinical Terms, SNOMED CT, pain assessment, patient-clinician communication, vector graphics, electronic health records, open-source tools

## Abstract

**Background:**

Accurate documentation of back pain is essential for effective diagnosis and treatment; however, conventional paper-based methods often lack precision and standardization, limiting their clinical utility. Digital pain drawings can improve consistency and integration; however, the literature continues to report variability in digital pain drawing methods, quantification approaches, body schemas, and implementation. Moreover, open access, anatomically labeled vector assets mapped to clinical terminology remain limited.

**Objective:**

This study aimed to develop an anatomically accurate, digitally segmented, open-source vector illustration of a sex-neutral human body (front and back views) for standardized back pain documentation and conduct a user-centered design validation and feasibility evaluation of its perceived usefulness, communication value, and visual acceptability.

**Methods:**

An interdisciplinary team used an iterative methodology informed by standard anatomical references to create the illustration, emphasizing anatomical precision, digital compatibility, and interoperability with medical coding systems. A survey-based design validation study (N=298) was conducted in the United Kingdom through the Prolific platform to evaluate perceived usefulness, confidence in identifying hypothetical pain regions (without an interactive drawing task or comparator), aesthetic appeal, and suggestions for improvement. Quantitative results were summarized descriptively with 95% CIs, and qualitative feedback guided final refinements. The finalized illustration was released under a CC BY 4.0 license.

**Results:**

Mean confidence ratings were 5.7 out of 7 (SD 1.2) for indicating pain regions using the illustration (95% CI 5.5-5.8) and 5.6 out of 7 (SD 1.2) for communicating pain with health care professionals (95% CI 5.5-5.8). Aesthetic ratings were more mixed (mean 4.3 out of 7, SD 1.5; 95% CI 4.1-4.5). User feedback highlighted the need for improved visual clarity, leading to adjustments in color contrast and anatomical proportions. Aesthetic concerns, including the removal of the areolae and changes to the facial features, were also addressed to enhance user comfort and neutrality.

**Conclusions:**

The resulting anatomically accurate vector illustration provides a feasible, open-source foundation for digital back pain documentation tools and may support patient-clinician communication and integration with electronic health records. The design validation study supports acceptability and perceived usefulness, but it does not establish superiority over existing body maps or objective accuracy of pain localization. By releasing it under a CC BY 4.0 license, we encourage broad adoption and community-driven improvements.

## Introduction

Back pain is one of the most common musculoskeletal conditions, affecting a substantial proportion of the global population [1,2]. It remains one of the leading causes of disability, contributing significantly to the global disease burden [3]. Accurate assessment and documentation of back pain are critical for effective diagnosis, treatment planning, and outcome monitoring. Traditionally, patients have used paper-based pain drawings to mark areas of discomfort, but these methods vary in quality and standardization and may lack the detail needed for advanced clinical integration [4]. Digital pain drawings have emerged as a more consistent and reliable alternative, enabling improved data integration, enhanced quantification of pain areas, and longitudinal tracking [5-7]. However, the literature still describes heterogeneity in pain drawing digitization methods, computerized quantification approaches, body schemas, and implementation workflows [5,8-11]. In this context, openly available, anatomically detailed vector illustrations with standardized labels and terminology mappings remain important enabling resources for broader clinical and research adoption.

Modern patient management benefits from highly standardized documentation to support accurate diagnosis, treatment planning, and outcome monitoring [12]. Historically, the interpretation of paper-based pain drawings has been inconsistent, and these analog methods have posed challenges for data integration [13]. In response, frameworks such as the Systematized Nomenclature of Medicine Clinical Terms (SNOMED CT) and *International Classification of Diseases, 11th Revision*, have helped enhance semantic interoperability and clinical data exchange, thereby improving the precision and utility of pain documentation [14,15]. The transition to digital pain drawings offers the potential for more accurate, scalable, and integrable data capture [5,16]; however, early digital solutions often lacked detailed anatomy or standardized labeling schemes, complicating integration with electronic health records (EHRs) and research databases [5]. Without clear links to widely recognized anatomical and clinical terminologies, such tools struggle to contribute effectively to epidemiological studies or decision support systems [5]. Modern advancements in usability and design encourage adoption among patients and clinicians, particularly when aligned with standardized terminologies [6,11,17].

Recent efforts have aimed to develop anatomically accurate digital representations of the human body, allowing for granular coding of pain locations and helping guide clinical decision-making [18,19]. By aligning these illustrations with standard terminologies, the data can be seamlessly incorporated into EHRs, clinical decision support systems, and large-scale research initiatives [20]. Open-source platforms have further gained traction in this domain, fostering community engagement, iterative improvement, and broad scalability for digital health tools [16,21]. Within pain assessment, open-source anatomically accurate illustrations facilitate customization for various patient populations and encourage continuous refinement of labeling accuracy and usability [6,13]. Over time, a robust, patient-centered ecosystem of clinically valid, standardized, and interoperable data for pain documentation can thus be developed [11,16].

To address the aforementioned limitations, we developed an anatomically accurate, sex-neutral human body vector illustration with digital labels specifically for back pain documentation. The illustration was created through several iterative design cycles by an interdisciplinary team. We then conducted a design validation and feasibility evaluation on a near-final version. This evaluation assessed perceived usefulness, aesthetic appeal, and potential support for patient-clinician communication, but it did not compare the illustration with other body maps or test objective marking accuracy. User feedback informed refinements that led to the final version now being released. The labels were premapped to SNOMED CT codes to support standardized documentation, although other coding systems could also be applied. By releasing this illustration under a CC BY 4.0 license, we aim to encourage broad adoption and community-driven enhancements. Feedback will continue to guide future iterations and inform the development of digital tools for back pain documentation, for which the illustration can serve as a foundational component.

## Methods

### Development of the Anatomically Labeled Vector Illustration

#### Collaborative Design and Development Process

The anatomically labeled vector illustration was developed through a tightly integrated, interdisciplinary effort involving illustrators, computer scientists, physicians (from anatomy, orthopedics, radiology, and integrative medicine), and psychologists. This collaboration ensured that the final illustration met both clinical requirements and usability standards. Serving as the primary interface for mapping patient-reported pain locations, the illustration had to balance anatomical precision with clarity and inclusivity.

Before the design work began, the team collectively defined core criteria to guide development:

- *Neutral representation*: a generalized, non–demographic-specific body outline to ensure accessibility across diverse patient groups
- *Anatomical accuracy*: detailed but approachable visuals to support orientation and reliable self-reporting by patients, including all relevant structures without oversimplification
- *Interoperability*: independently vectorized and hierarchically structured anatomical components (eg, limbs to phalanges) incorporating clinical landmarks such as vertebra C7 to support integration with medical systems
- *Versatility*: design adaptable for various clinical, research, or corporate software environments, including customizable layers and labeling schemes

To ensure anatomical correctness throughout, an anatomist continuously reviewed intermediate designs. The illustration originated from manual tracing of standard anatomical references [22-25] aligned in front and back views using Adobe Illustrator. Components such as bones, soft tissue, and clinical landmarks were separated into dedicated layers, enabling modular refinement and review.

#### Iterative Review Process

##### Overview

To ensure anatomical accuracy and clinical applicability, the design process followed a structured, iterative review cycle. Each iteration began with a comprehensive evaluation of the illustration by the interdisciplinary team, including an anatomist who scrutinized the bone and soft tissue outlines against standard anatomical references. Their expertise ensured that proportions, spinal curvature, and skeletal alignments were anatomically accurate and clinically consistent. Feedback from these reviews was then systematically analyzed and integrated into the next version.

##### First Review Cycle

The initial review focused on structural alignment and proportions. A misalignment in the vertebrae between the front and back views was identified, caused by perspective effects in the reference images. This issue was corrected in the second iteration by aligning the vertebrae across views and refining proportions, including adjustments to the height of the pelvis, ankles, axillae, and other key regions. Soft tissue details were also enhanced to aid orientation and improve distinction between the front and back views, incorporating features such as the costal arch, inguinal fold, scapular protrusions, elbows, knees, wrists, and finer details such as skin folds and nails.

##### Second Review Cycle

Subsequent refinements addressed consistency and visual clarity. The clinical landmarks were matched to the final positions of the skeletal features, and the vertebrae were redrawn to improve proportionality. Additional visual elements such as shading and subtle color cues were introduced to preview the use case of the illustration and improve clarity in distinguishing anatomical structures. The silhouette of the body was further refined to ensure a cleaner and more accurate representation.

##### Third Review Cycle

In the prefinal review, a need for better spatial distinction between anterior and posterior structures was identified. This issue was particularly pronounced in the thorax, where the 3D structure made it challenging to differentiate between the anterior and posterior aspects of the ribs. To resolve this, the posterior sections of the ribs and select bones were dimmed, improving spatial clarity and overall distinction.

##### Fourth Review Cycle and Standardization

In the final iteration, the components of the illustration were systematically named according to scientific nomenclature, ensuring compatibility with medical coding systems such as SNOMED CT (see the Labeling Approach and SNOMED CT Mapping section). The vector elements were hierarchically grouped and organized into layers within the SVG file to facilitate standardized documentation and seamless integration with EHR systems. The finalized illustration underwent thorough checks to confirm alignment with the initial design criteria and interoperability goals.

### Final Revisions Based on User Feedback

In addition to the iterative reviews conducted by the interdisciplinary team, we incorporated insights from our user study to address key aesthetic concerns (Figure 1). The main changes included removing the prominently drawn areolae, redesigning facial features to reduce an “uncanny” appearance, and adjusting contrast and color palettes to improve visual clarity. We also refined minor aspects of body proportions in response to participant feedback on overall aesthetics.

**Figure 1.**
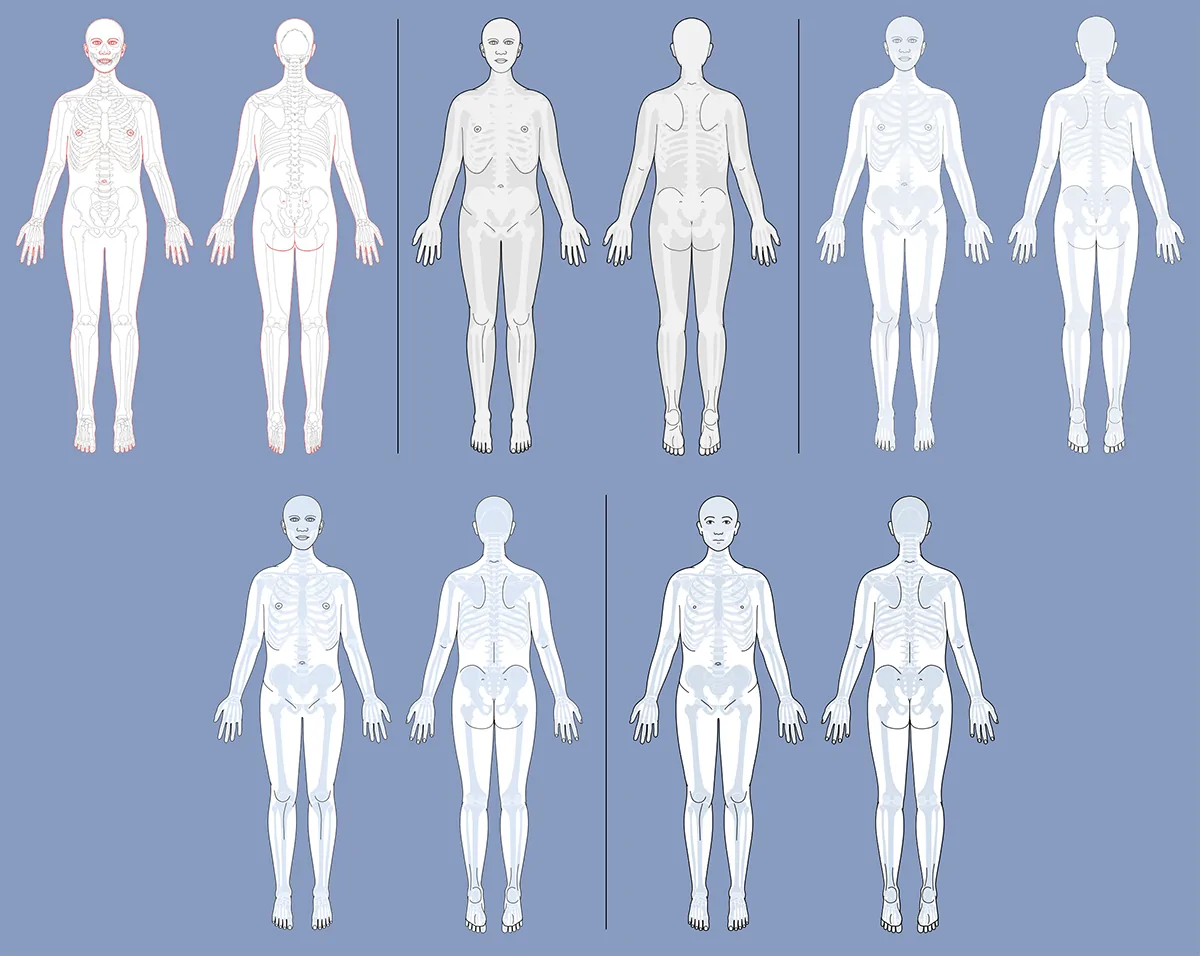
Design evolution of the anatomical vector illustration across 5 iterations. In the top row (left to right), the first 3 iterations show progression from the initial prototype with detailed facial features and high-contrast outlines to adjustments to skeletal proportions, shading, and anatomical accuracy. In the bottom row (left to right), the fourth iteration was used in the user study, followed by the final version featuring improved spatial clarity between the anterior and posterior views, neutral aesthetics, and refinements for enhanced clinical applicability. These refinements were informed by structured expert reviews and user feedback from the evaluation study.

### Internal Vector Structure and Layering

The vector illustration is structured in a clear, hierarchical way to facilitate easy manipulation and identification of body regions for pain drawing applications. At the highest level, the illustration is divided into main groups representing anatomical views—anterior (“bones-ant”) and posterior (“bones-pos”). Each view contains subgroups for distinct anatomical regions, such as “columna” (spinal column), “cranium,” “coxa” (hip region), and “superius” (upper extremities). Within each region, individual bones or vertebrae are defined as separate, clearly labeled elements within the SVG file (eg, “bones-pos-columna-C01” for cervical vertebra C01). This organized layering allows users or software developers to quickly access and highlight specific anatomical structures programmatically, greatly enhancing usability and interoperability in digital health applications.

### Labeling Approach and SNOMED CT Mapping

The labeling of anatomical structures in the SVG illustration follows a systematic and standardized naming convention, ensuring clear identification and consistent referencing across applications. Each SVG element has a unique identifier directly corresponding to a specific anatomical structure. To further facilitate semantic interoperability, we mapped these identifiers to standardized clinical terminology codes from SNOMED CT. This mapping is provided in a structured JSON file, where each SVG element ID (eg, “bones-pos-clavicula-sin”) is directly associated with its corresponding SNOMED CT concept identifier (eg, 720617006). This structured mapping simplifies integration with EHRs and clinical documentation systems, enabling precise and unambiguous representation of anatomical locations in digital health and clinical informatics applications.

### Open-Source Release

The final vector illustration was released under a CC BY 4.0 [26] license in the GitHub repository *Open Pain Illustration* [27]. Open-source availability encourages community engagement for further refinement and widespread adoption.

### User Study

#### Study Objectives

The user study was conceptualized as a design validation and feasibility evaluation with 4 primary objectives. First, it sought to assess user confidence in using the illustration to represent back pain. Second, it aimed to evaluate the perceived usefulness of the illustration in facilitating communication about back pain with health care professionals. Third, it examined the aesthetic appeal and visual acceptability of the illustration. Finally, it gathered actionable suggestions to improve the illustration’s usefulness and visual design. The study was not designed to compare the illustration with existing body maps or measure objective pain marking accuracy.

#### Study Design and Procedures

##### Participants

Participants were recruited via Prolific [28] using Prolific’s representative sampling functionality, which aims to approximate representativeness based on sex, age, and ethnicity according to the simplified UK census demographics [29]. Inclusion criteria were adults aged 18 years and older regardless of their history of back pain. Because recruitment took place through an online platform, participation required internet access and sufficient digital literacy to complete a survey. All participants provided informed consent before beginning the study. The recruitment goal was 300 participants to ensure a sample size large enough to provide robust descriptive statistics and meaningful insights into population characteristics. Two participants were excluded due to technical issues, and rerecruitment was deemed unnecessary as the final sample of 298 participants remained representative of the target population. Participants received £0.75 (£1=US $1.29 as of October 31, 2024) for completing the study.

##### Procedure

Participants were shown the developed anatomical vector illustration from the fourth review cycle (Figure 1) and instructed to consider how they would use it to indicate areas of back pain. Those with current back pain were asked to reflect on how they would depict their actual pain, whereas those without back pain were instructed to imagine having back pain and how they would illustrate it. This task was conceptual rather than interactive: participants did not draw directly on the illustration, no alternative body map was shown, and no objective marking task was scored for anatomical accuracy. Following this, all participants completed an online survey designed to evaluate their confidence in depicting and communicating pain, as well as their perceptions of the chart’s aesthetics. The survey also included open-ended questions to gather qualitative feedback.

##### Survey Measures

The survey included both closed-ended and open-ended questions. Closed-ended questions were rated on a 7-point Likert scale, whereas open-ended responses allowed participants to provide detailed feedback. The survey assessed confidence in accurately representing back pain using the anatomical vector illustration (referred to as the “body chart” in the survey), confidence in using the illustration to communicate pain to health care professionals, and the aesthetic appeal of the illustration. Open-ended responses captured suggestions for improving its usefulness and aesthetics. Additionally, demographic information, including age, sex assigned at birth, gender identity, and back pain history, was collected. A full version of the survey is provided in Multimedia Appendix 1.

### Data Analysis

Quantitative data were analyzed using descriptive statistics. For each rating outcome, 2-sided 95% CIs for the proportion of participants providing a positive rating, defined as a score of 5 to 7, were calculated using the Wilson score method. Two-sided 95% CIs for means were calculated using the Student *t* distribution. No hypothesis tests were performed. Qualitative feedback on aesthetics and the perceived ability to indicate pain was examined through thematic analysis facilitated by the OpenAI *o1-preview* language model [30]. Participants’ open-ended responses were analyzed thematically to uncover key patterns, recurring themes, and actionable suggestions. Due to the large volume of textual data, the language model was used to assist in organizing and summarizing the feedback. Two separate prompts were designed for the analysis of each open-ended question (Multimedia Appendix 2), enabling the model to generate structured outputs, including recurring themes, the number of participants mentioning each theme, representative quotes, and participant identifiers for manual review. To ensure the validity of the AI-generated results, the participant identifiers were manually reviewed by one of the authors, confirming that the themes accurately reflected the responses, with no mismatches identified.

### Ethical Considerations

This study was conducted in accordance with ethical standards. The study synopsis was submitted to the Ethics Committee of Zurich, Switzerland, which determined that the study did not fall under the regulation of the Swiss Human Research Act (ethics ID 2022-01475). Participation was voluntary, and all participants provided electronic informed consent before beginning the survey. Survey data were collected anonymously, with no personal identifiers recorded, and securely stored.

## Results

### Participant Demographics

A total of 298 participants were included in the analysis. The mean age of the sample was 46.2 (SD 15.4) years (95% CI 44.4-47.9). Of 298 participants, 154 (51.7%) were assigned female at birth, and 144 (48.3%) were assigned male at birth. Regarding back pain history, 51.7% (154/298) of the participants reported current back pain, 31.5% (94/298) had a history of back pain, and 16.8% (50/298) reported no back pain.

Detailed demographic characteristics are summarized in Table 1. In total, 0.7% (2/298) of the participants, who identified as female, were assigned male at birth. No participants who identified as male were assigned female at birth. A total of 5% (15/298) of the participants did not provide any gender description. Education and race and ethnicity distributions are also shown in Table 1.

**Table 1.** Participant demographics (N=298).

Characteristics	Values
Age (y), mean (SD; 95% CI)	46.2 (15.4; 44.4-47.9)
Sex assigned at birth, n (%)	
Female	154 (51.7)
Male	144 (48.3)
Back pain history, n (%)	
Current back pain	154 (51.7)
History of back pain	94 (31.5)
No back pain	50 (16.8)
Gender identity relative to sex assigned at birth, n (%)	
Sex and gender identity concordant	281 (94.3)
Identified as female; assigned male at birth	2 (0.7)
Identified as male; assigned female at birth	0 (0)
No gender description provided	15 (5.0)
Educational level, n (%)	
Bachelor’s degree	96 (32.2)
Master’s degree or higher	58 (19.5)
Upper secondary qualifications without university access	80 (26.8)
University entry qualification	31 (10.4)
Lower secondary qualifications	21 (7.0)
Postsecondary education below Bachelor’s level	10 (3.4)
No formal qualifications	2 (0.7)
Race and ethnicity, n (%)	
Asian	22 (7.4)
Black	11 (3.7)
Latino	1 (0.3)
Middle Eastern	4 (1.3)
White	247 (82.9)
Other	5 (1.7)
Preferred not to answer	5 (1.7)
Selected multiple racial categories	3 (1.0)

### Confidence in Indicating Pain

Participants’ confidence in accurately indicating their back pain on the body chart varied across the Likert scale. Overall, 85.9% (256/298) of the participants (95% CI 81.5%-89.4%) reported a positive level of confidence (scores>4). The mean rating was 5.7 out of 7 (SD 1.2; 95% CI 5.5-5.8; N=298). In a descriptive subgroup analysis, participants with current back pain had a mean rating of 5.8 (95% CI 5.6-6.0; n=154), whereas participants in the “no back pain” category had a mean rating of 5.3 (95% CI 5.0-5.6; n=50). Participants in the “history of back pain” category (n=94) were not included in this contrast. No hypothesis test was performed. Only 5.7% (17/298) of the participants reported low confidence (scores<4), indicating that low confidence ratings were uncommon (Figure 2).

**Figure 2.**
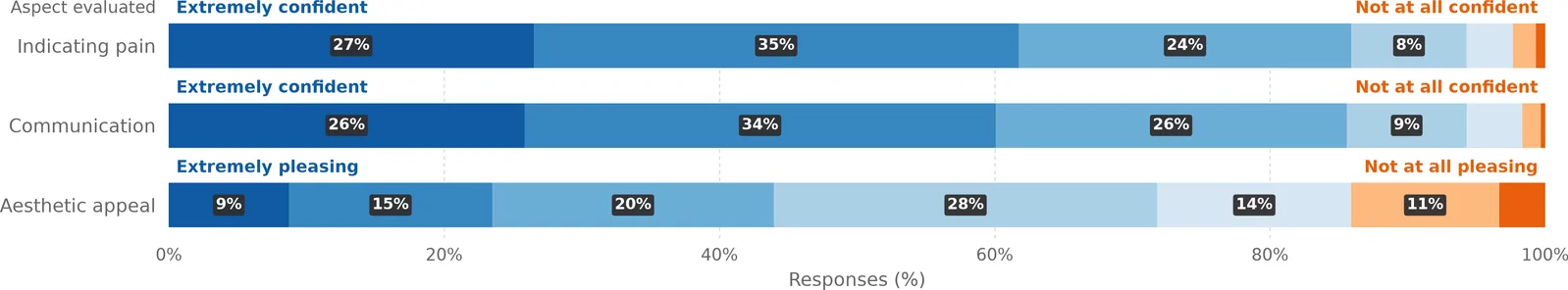
Stacked horizontal bar chart showing Likert-scale response distributions to the 3 survey items: confidence in indicating pain, communication with health care professionals, and aesthetic appeal.

### Communication With Health Care Professionals

Most participants felt confident that the body chart illustration would aid communication with health care professionals. Similar to the pain indication confidence ratings, 85.6% (255/298) of the participants (95% CI 81.1%-89.1%) expressed positive confidence in this ability (scores>4), with a mean score of 5.6 out of 7 (SD 1.2; 95% CI 5.5-5.8). Only 5.7% (17/298) of the respondents reported low confidence in this ability (scores<4), as shown in Figure 2.

### Aesthetic Appeal

The illustration’s aesthetic appeal received mixed feedback: 44% (131/298) of the participants (95% CI 38.4%-49.6%) rated it positively (>4), 27.9% (83/298) rated it neutrally (4), and 28.2% (84/298) rated it negatively (<4). This variability resulted in a lower mean score of 4.3 out of 7 (SD 1.5; 95% CI 4.1-4.5), indicating room for improvement in the visual design (Figure 2).

### Summary of Feedback on Improvement Suggestions

Participants provided numerous suggestions to improve functionality and visual appeal. Although this study only presented a static illustration without any interactive features or drawing tools, many participants proposed enhancements that they would find useful in a fully functional version. Common suggestions for improving functionality included the ability to zoom or enlarge the image, add labels or anatomical landmarks, replace the skeletal view with muscular structures, and use color coding to indicate pain type or intensity. Some participants also expressed interest in additional perspectives such as side views and the ability to depict radiating or moving pain. Aesthetic feedback focused on modifying facial features, redesigning or removing nipples, adjusting body proportions, and optionally providing sex-specific versions. These insights informed final refinements and will guide future iterations. A detailed breakdown is available in Multimedia Appendix 3.

## Discussion

### Principal Results

Our findings should be interpreted as results of a design validation and feasibility evaluation rather than as evidence of comparative or clinical effectiveness. Because the user study did not include an alternative body map or an objective pain marking task, it cannot determine whether the positive confidence ratings were attributable to qualities specific to this illustration or reflected more general comfort with body mapping. Within those limits, the findings suggest that an anatomically accurate, open-source vector illustration for back pain documentation was acceptable to many users and was perceived as potentially useful for identifying and communicating pain regions while also revealing areas that may benefit from further refinement. A substantial proportion of participants reported confidence in their ability to use the illustration, suggesting that its level of detail and clarity may be appropriate for many users. Individuals currently experiencing back pain reported slightly higher confidence than participants in the “no back pain” category; because this was a descriptive subgroup observation, it should be considered hypothesis generating rather than confirmatory. However, user feedback underscored the need for future iterations to address varying needs, such as additional labeling options, alternative views, and aesthetic adjustments.

Building on these insights, the qualitative feedback highlighted several opportunities to enhance both functionality and usefulness. Participants advocated for greater precision and flexibility, such as enlarging the image, enabling zoom, adding annotations, or including multiple viewpoints (eg, side views) to capture pain that radiates or shifts. These suggestions align with broader literature emphasizing usability and adaptability as key drivers of patient engagement with digital health tools [6,11,16,17]. Calls for more nuanced and realistic representations, such as incorporating muscles, also reflect the evolving understanding of how anatomical accuracy can enhance the assessment and communication of musculoskeletal pain [5].

Aesthetically, participants revealed a tension between clinical neutrality and visual appeal. While some felt that aesthetics was secondary to the tool’s medical purpose, others desired features that would humanize the figure—less unsettling facial features, optional sex-specific versions, realistic body proportions, and color enhancements. This variability underlines the importance of designing medical visualization tools that are both functionally rigorous and sensitive to users’ comfort and emotional responses, a consideration increasingly recognized in medical illustration and patient-centered design research.

Back pain is common among older adults [1-3]. Older adults and people with low digital literacy may face barriers when using digital interfaces, although the nature of these barriers may differ. Prior research indicates that digital pain drawings can be reliable [13] and that pain drawing interfaces should be tailored to users with physical or cognitive impairments [5]. In our study, the mean participant age was 46.2 (SD 15.4) years, and self-reported confidence regarding use of the illustration was generally high. However, because online recruitment required internet access and sufficient baseline digital literacy, these findings may not generalize to older adults or patients with low digital literacy. Future usability testing should therefore specifically include patients with low digital literacy, limited device access, or a need for assisted completion to assess accessibility, usability, and reliability in clinical and at-home contexts.

From a clinical informatics perspective, the integration of anatomical labels aligned with coding standards such as SNOMED CT represents a significant step toward interoperability. By ensuring that the illustrated regions can be mapped onto recognized clinical terminologies, we opened avenues for integrating patient-generated pain data into EHRs, large-scale research databases, and decision support systems. This interoperability can ultimately enrich the landscape of clinical data, supporting more consistent documentation, outcome tracking, and evidence-based decision-making in back pain management [14,15,20].

### Limitations and Future Directions

Notwithstanding these strengths, our study has limitations. Although we recruited a relatively large sample (N=298) with diverse backgrounds, online crowdsourced participants may not fully represent clinical populations or individuals with severe mobility impairments, non-English speakers, patients in specific health care contexts, or people with low digital literacy and limited internet or device access. This limitation is particularly relevant for future at-home use, where successful adoption will require interfaces, instructions, and support options that work for patients with varying digital skills. Additionally, our evaluation relied primarily on self-reported measures of confidence and perceived usefulness rather than longitudinal assessments or clinical outcomes. Future research could involve clinical trials that track how using such illustrations influences communication in consultations, diagnostic accuracy, treatment adherence, or patient satisfaction over time and should include dedicated assessments for low–digital literacy users. Furthermore, the user study included additional questions that will be explored in a future publication.

Another important limitation is that the current illustration includes only anterior and posterior views. While some participants expressed interest in having additional perspectives, particularly lateral or side views, to capture certain types of radiating or localized back pain, we have no immediate plans to add such views at this stage. Future community-driven adaptations or subsequent versions of the illustration could address this need by expanding the anatomical perspectives, but this remains outside the current scope of development. Consequently, clinicians and researchers should be aware of potential gaps in capturing pain that extends or radiates laterally.

While participants’ positive self-reported confidence suggests promise for the current illustration, it does not guarantee objective accuracy, superiority over existing body maps, or improved clinical decision-making. We acknowledge that future investigations must move beyond self-reports to assess whether patients’ markings on our illustration consistently match clinical findings or imaging results, ideally in studies with comparator body maps and predefined accuracy metrics. To that end, we are currently developing a dedicated digital tool that incorporates this body illustration and can be used on various devices (eg, personal smartphones and clinic-based tablets). This tool will allow patients to interactively mark their pain regions in real time and submit those markings securely to their health care professionals. By leveraging the SNOMED CT codes embedded within each body region, we intend to integrate these patient-drawn pain diagrams into EHR systems. Such direct linkage will enable clinicians to compare reported pain locations and severities against diagnostic imaging or clinical assessments, offering a more robust evaluation of the tool’s accuracy than subjective confidence ratings alone.

Evaluating the long-term and real-world impact of the illustration likewise remains a vital next step. Our future studies will embed the digital pain reporting tool seamlessly into clinical workflows, capitalizing on standard health data exchange protocols to transmit pain diagrams to the appropriate clinicians at the point of care. For example, patients in a pilot clinic setting may draw their pain areas at home or during triage, with these data flowing directly into the clinic’s electronic system (via Fast Healthcare Interoperability Resources– or Health Level Seven version 2–based messaging) to alert clinicians before the consultation. Such integration will allow for longitudinal tracking of how pain evolves over time, comparisons with clinical outcomes (eg, functional scores and medication use), and formal assessment of whether digital body mapping leads to better targeted interventions. By capturing these objective metrics in routine practice and pairing them with patient-reported data, we can more definitively establish whether our anatomically accurate, open-source illustration and its future associated digital application foster improvements in back pain care.

### Conclusions

The anatomically accurate, open-source vector illustration for back pain documentation represents a feasible foundation for future digital health tools. In this design validation study, the illustration showed promise in supporting patient confidence, communication with clinicians, and integration into EHRs through alignment with coding standards such as SNOMED CT. However, the present results do not demonstrate superiority over existing body maps or objective improvements in pain localization accuracy. User feedback highlights opportunities for refinement, such as adding muscular details; optional sex-specific versions; and features such as zooming, annotations, and multiple perspectives. Moreover, future tools incorporating this illustration could further enhance usefulness by addressing functionality needs such as capturing radiating pain and offering customizable options. Iterative improvements to both the illustration and the tools that leverage it could promote broader adoption, improve standardization, and better meet diverse user needs. Future research should evaluate how the illustration and these tools impact clinical workflows and patient outcomes, ensuring their effectiveness in varied health care settings.

## Supplementary material

10.2196/75737 Multimedia Appendix 1 Survey questions used in the user study.

10.2196/75737 Multimedia Appendix 2 Prompt templates used for the thematic analysis of open-ended survey responses on pain drawing accuracy and aesthetic feedback.

10.2196/75737 Multimedia Appendix 3 Detailed results from the thematic analysis of participant feedback, including identified themes, representative quotes, and participant counts.

## Abbreviations

EHR: electronic health record
SNOMED CT: Systematized Nomenclature of Medicine Clinical Terms
