# Supplementary material for: Open-Source Anatomical Illustration for Digital Back Pain Documentation: Development and Feasibility Evaluation
Source: JMIR Med Inform. 2026 Oct 15;14:e75737. doi: 10.2196/75737 (PMC13671485; doi:10.2196/75737)
Supplement: Supplementary file 1 [file medinform-v14-e75737-s001.pdf]

Multimedia Appendix 1

Survey Questions (that were included in this paper)

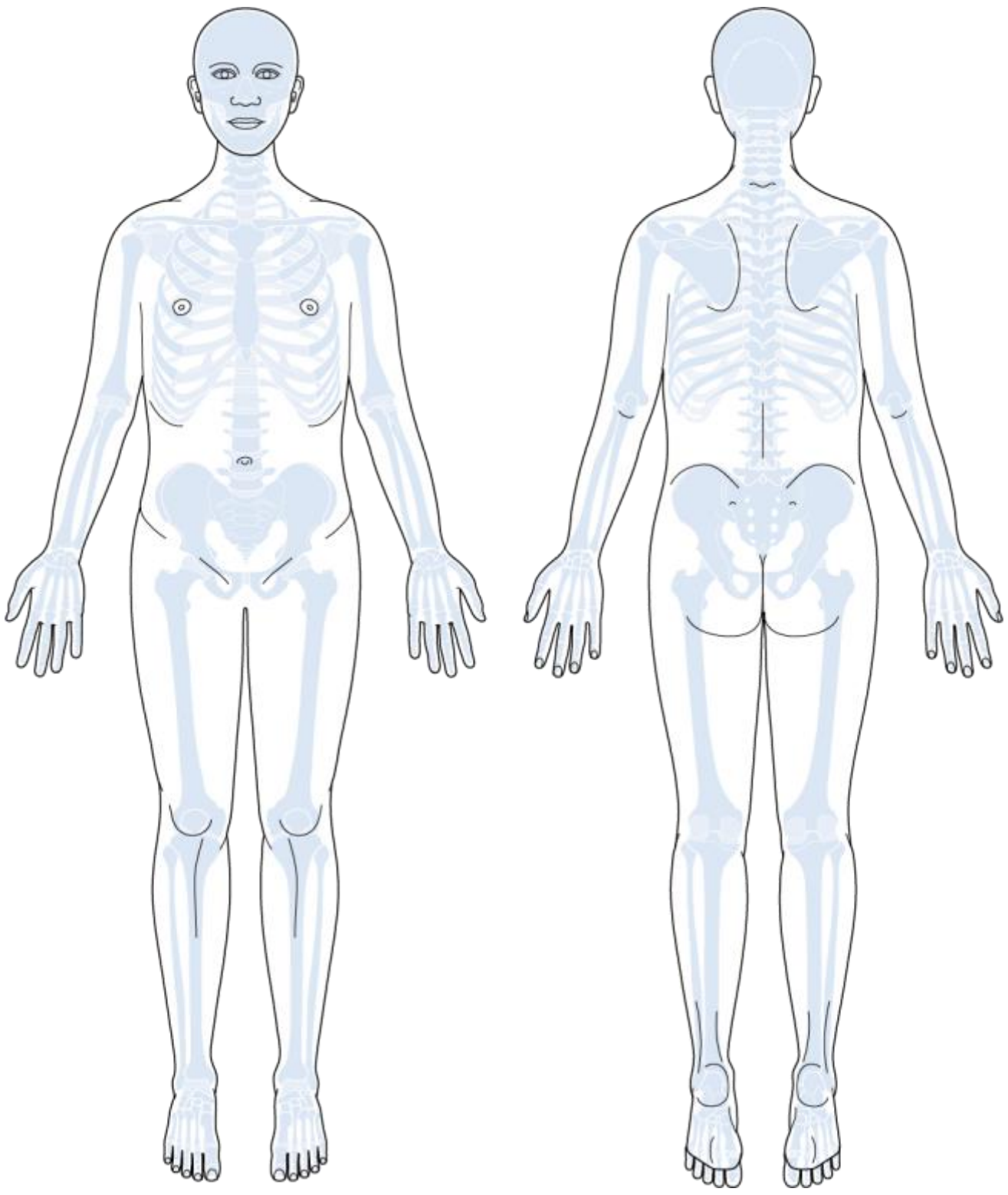

This pain drawing body chart is designed for people with back pain. If you don't have back pain, please imagine you do.

Question	Answer Options
**How confident are you that you would be able to accurately draw your back pain on this body chart?**	A1 = "Not at all confident" to A7 = "Extremely confident"
**If you could change anything about this body chart to help you draw your pain more accurately, what would it be?**	Comments (optional)
**How confident are you that using this body chart to draw your back pain would help you communicate your pain to a healthcare professional?**	A1 = "Not at all confident" to A7 = "Extremely confident"
**How aesthetically pleasing do you find this body chart?**	A1 = "Not at all pleasing" to A7 = "Extremely pleasing"
**If you could change anything about this body chart to make it more aesthetically pleasing, what would it be?**	Comments (optional)
**What is your age in years?**	Years
**Do you currently experience back pain?**	Yes<br>No
**Have you experienced back pain in the past?**	Yes<br>No
**If yes, for how long have you experienced back pain?**	Years
	Months
**In which country do you currently reside?**	drop-down list
**What sex were you assigned at birth?**	F = Female<br>(?) = Intersex

	M = Male<br>Empty = I prefer not to answer
**How do you currently describe your gender?**	Please specify<br>Empty = I prefer not to answer
**Which of the following is the highest qualification or level you earned or completed?**	A1 = No formal qualification<br>A2 = Lower secondary qualification<br>A3 = Upper secondary qualification without university access<br>A4 = University entry qualification<br>A5 = Post secondary below bachelor’s level<br>A6 = Bachelor’s level<br>A7 = Master’s level and above
**When thinking about physical attributes usually ascribed to race, which of the following general labels describe how you would describe yourself racially: (mark ALL that apply)**	Asian
	Black
	Indigenous, Aboriginal, or First Nations
	Latino or Hispanic
	Middle Eastern
	White
	Other, please specify:______
	I prefer not to answer
