# Supplementary material for: Open-Source Anatomical Illustration for Digital Back Pain Documentation: Development and Feasibility Evaluation
Source: JMIR Med Inform. 2026 Oct 15;14:e75737. doi: 10.2196/75737 (PMC13671485; doi:10.2196/75737)
Supplement: Supplementary file 2 [file medinform-v14-e75737-s002.pdf]

Multimedia Appendix 2

Prompts for Thematic Analysis

Prompt for Ability to Draw Pain Improvements

```
We designed a vector illustration to help patients indicate their back pain. To
gather feedback, we conducted a representative study with 298 participants from
the UK, asking:

“If you could change anything about this body chart to help you represent your
pain more accurately, what would it be?”

Below are their responses. Please analyze and:

1. Identify recurring themes or common topics in the feedback.
2. Provide the number of participants who mentioned each topic.
3. Include 1-2 representative quotes from participants for each topic.
4. For each theme or topic identified, return the list of all participants that
belong to that topic for manual review.

**Please present your response in the following standardized format:**

For each theme or topic identified:

- **Theme/Topic Name**:
- **Number of Participants**: [Number]
- **Representative Quotes**:
  - "Quote from Participant"
  - "Another Quote from Participant"
- **Full List of Participants**: PXXX, PXXX, ...

Participants' responses:
PXXX: ...
PXXX: ...
...
```

Prompt for Aesthetic Improvements

```
We designed a vector illustration to help patients indicate their back pain. To
gather feedback, we conducted a representative study with 298 participants from
the UK, asking:

"If you could change anything about this body chart to make it more aesthetically
pleasing, what would it be?"

Below are their responses. Please analyze and:

1. Identify recurring themes or common topics in the feedback.
2. Provide the number of participants who mentioned each topic.
3. Include 1-2 representative quotes from participants for each topic.
4. For each theme or topic identified, return the list of all participants that
belong to that topic for manual review.

**Please present your response in the following standardized format:**

For each theme or topic identified:

- **Theme/Topic Name**:
- **Number of Participants**: [Number]
- **Representative Quotes**:
  - "Quote from Participant"
  - "Another Quote from Participant"
- **Full List of Participants**: PXXX, PXXX, ...

Participants' responses:
PXXX: ...
PXXX: ...
...
```
