# Supplementary material for: Open-Source Anatomical Illustration for Digital Back Pain Documentation: Development and Feasibility Evaluation
Source: JMIR Med Inform. 2026 Oct 15;14:e75737. doi: 10.2196/75737 (PMC13671485; doi:10.2196/75737)
Supplement: Supplementary file 3 [file medinform-v14-e75737-s003.pdf]

Multimedia Appendix 3

Detailed Feedback on Ability to Draw Pain Improvements and Aesthetic Improvements

Feedback on Ability to Draw Pain Improvements

Participants provided several suggestions to enhance the body chart for more accurate pain representation. The most frequently mentioned themes were:

Image Size and Zoom Functionality: Seventeen participants expressed the need for a larger image or the ability to zoom in for more precise pain indication. For instance, Participant P011 stated, "I would make the image larger so I would be able to more accurately draw exactly where the pain in my back is," and P281 suggested, "Make more detailed, closer in to help identify pain area more accurately."

Addition of Labels or Annotations: Thirteen participants recommended adding labels or anatomical landmarks to the diagram to assist in pinpointing pain locations. Participant P034 remarked, "Label the back areas to make it more easier to pinpoint where the pain is," while P038 noted, "It would be good to see a labelled diagram to show different points of back so easier to describe to medical professionals."

Inclusion of Muscles in the Diagram: Fourteen participants suggested adding muscular anatomy or replacing the skeleton with muscles to better represent soft tissue pain. For example, P023 said, "Maybe add in pictures of the muscles," and P101 proposed, "I would remove the skeleton and replace it with muscle groups."

Use of Color to Indicate Pain Severity or Type: Eleven participants proposed incorporating colors to denote different pain intensities or types. P005 suggested, "Possibly having a colour chart to guide people how to colour severity," and P016 added, "Maybe if I could use colors to show the intensity? Like red for intense pain, amber for dull pain."

Provision of Male and Female Body Charts: Six participants preferred sex-specific body charts. P009 stated, "Use a female body model as well as a male," and P290 echoed, "Two versions of the chart, one male and one female."

Additional Body Views: Six participants requested side views or additional perspectives to better represent pain locations. P116 commented, "Only thing I can think of would be a side-on diagram in addition to the front and back views," and P150 suggested, "A side view to indicate the depth of the pain."

Different Body Shapes/Sizes: Four participants suggested reflecting individual differences with various body shapes. P014 proposed, "Have different sized charts for different body types," and P018 asked, "Maybe different-shaped bodies?"

Ability to Indicate Radiating or Moving Pain: Three participants wanted options to depict pain that radiates or moves. P007 expressed, "The ability to indicate if it radiates further," and P040 stated, "I would like the opportunity to explain that my pain moves around."

Notably, 83 participants indicated that they would not change anything about the chart, stating that it was sufficient for their needs. Additionally, 54 participants did not provide any suggestions.

Feedback on Aesthetic Improvements

Participants also provided feedback on the aesthetic aspects of the body chart, highlighting areas for enhancement. Key themes were:

Facial Features Being Unsettling: Twenty-two participants found the facial depiction to be unsettling or "creepy." For example, P024 commented, "Change the faces, they're quite disturbing," and P045 stated, "I think the person looks a little creepy, I would like a nicer looking person."

Removal or Modification of Nipples: Eleven participants suggested removing or altering the depiction of nipples, finding them distracting or unnecessary. P015 remarked, "Remove the nipples," and P264 added, "Remove the nipples they are creepy looking."

Addition of Color: Twenty-one participants recommended adding color to make the chart more visually appealing. P007 suggested, "Colour maybe," and P090 noted, "I think it would be better in colour."

Inclusion of Labels or Markings: Eight participants proposed adding labels or markings to enhance clarity. P018 asked, "Maybe some labels?" and P272 suggested, "Labels on key points of the body or colour coding to separate systems."

Adjustments to Body Proportions or Anatomy: Six participants recommended modifying the body proportions or anatomical features for accuracy and realism. P008 pointed out, "The pelvic bones need to be moved lower down," and P072 mentioned, "The butt is a little too narrow and the colour of the skeleton is a bit too washed out."

Provision of Male and Female Versions: Eleven participants expressed a desire for sex-specific illustrations. P028 suggested, "Male and female versions... for us as appropriate," and P290 requested, "A female version."

Addition of Hair or Other Features: Seven participants recommended adding hair or other distinguishing features to make the figure appear more lifelike. P029 said, "Maybe add features like hair," and P274 added, "Give it some hair."

Adjustments to Skeleton Visibility: Three participants found the skeleton overlay distracting and suggested its removal or modification. P137 stated, "Don't have a picture of the skeleton," and P132 commented, "It's a bit unsettling seeing both the skeleton and the skin."

Color Scheme Adjustments: Four participants suggested adjusting the color scheme to enhance visual appeal. P050 noted, "Perhaps the colours could be a little less cold," and P161 said, "I'm not sure why, but the blue colour is off-putting."

Perception that Aesthetics Are Not Crucial: Nine participants felt that the aesthetic appeal was not essential for the chart's purpose. P095 noted, "I don't think it needs to be aesthetically pleasing. I would be content to use this as a neutral body chart," and P123 stated, "It doesn't need to be aesthetically pleasing as it's medical."

Again, 89 participants were satisfied with the current design for the intended purposes, and 78 offered no suggestions.
